# Trans-thoracic echo evaluation before and during noninvasive ventilation

**DOI:** 10.1186/cc10695

**Published:** 2012-03-20

**Authors:** L Vetrugno, M Costa, C Centonze, N Langiano, M Rojatti, G Della Rocca

**Affiliations:** 1 University Hospital of Udine, Italy

## Introduction

Over the last decade noninvasive ventilation (NIV) gained the dignity of first-line intervention for acute lung injury (ALI) and acute respiratory distress syndrome (ARDS) in the ICU. Its great interest is based on a lower complications rate compared with traditional invasive ventilation. However, the NIV application, although less invasive, cannot ignore its hemodynamic effect over the patient. This study evaluates the NIV effects on the left ventricle in terms of systolic and diastolic function through trans-thoracic echocardiography (TTE). We also try to obtain a preload value index equivalent of flow time corrected (FTc).

## Methods

Thirteen patients admitted to our ICU with ALI/ARDS underwent TTE before and during NIV. NIV was set as a 1 hour cycle with 5 to 7 cmH_2_O of PEEP and 5 to 7 cmH_2_O of pressure support ventilation. During NIV for a better patient compliance a continuous i.v. infusion of remifentanil was used (range 0.03 to 0.05 μg/kg/minute). At baseline (T0 = before NIV) and after 30 minutes of NIV (T1), the following data were recorded: respiratory - RR, SaO_2_%, PaO_2_, PaCO_2_, pH, BE, and HCO_3_^-^; and cardiac - heart rate (HR), arterial blood pressure (systolic, diastolic and media), diastolic and systolic volume (EDV, ESV), ejection fraction (EF), stroke volume (SV), velocity time integral (VTI), FTc, E wave, deceleration time (Dt), A wave, ventricular flow propagation velocity (Vp).

## Results

From T0 to T1 the following changes with Wilcoxon matched pairs test were statistically significant (*P *< 0.05*). PaO_2 _(94 to 123 mmHg*), SaO_2 _(87 to 97%*) and PaO_2_/FiO_2_, RR (37 to 28/minute*). At T0, EF was >55% in seven patients and <55% in six patients. In the group with EF <55% (T0) the EF increased at T1 (42 to 52%*). Dt significantly increased from T0 to T1 (182 to 198 cm/second*). No significant changes were observed in VTI, E/A ratio, Vp, and E/Vp ratio, from T0 to T1.

## Conclusion

Our study suggests that NIV improves cardiac function in patients with reduced EF, positioning the patients to a more favorable point of the Frank Starling curve. In these patients we also showed an increase in FTc that seems to be affected by either preload or afterload reduction.
